# Association of selected *ELAC2*, *MSR1*, *RNASEL* and *KLK3* polymorphisms with prostate cancer susceptibility in a Hungarian cohort

**DOI:** 10.3389/pore.2026.1612550

**Published:** 2026-08-28

**Authors:** Sebestyen Kovacs, Renata Szalai, Laszlo Barati, Kinga Hadzsiev

**Affiliations:** 1 Department of Urology, Medical School,University of Pécs, Pécs, Hungary; 2 Department of Medical Genetics, Medical School, University of Pécs, Pécs, Hungary

**Keywords:** Hungarian, polymorphism, prostate cancer, RNASEL, rs486907

## Abstract

**Background:**

Prostate cancer (PCa) remains a leading malignancy among men worldwide, with inherited genetic susceptibility playing a pivotal role in its pathogenesis. Although numerous single nucleotide polymorphisms (SNPs) within immune response, inflammatory, and apoptotic pathways have been linked to PCa risk, these associations often exhibit substantial inconsistency across diverse populations. This study aimed to evaluate the association of selected polymorphisms within the *RNASEL*, *ELAC2*, *MSR1*, and *KLK3* genes with PCa susceptibility in a Hungarian cohort.

**Methods:**

This case-control study included 103 histologically confirmed prostate cancer patients and 103 healthy controls recruited at the University of Pécs Department of Urology. Genotyping of selected polymorphisms was performed by PCR and Sanger sequencing at the Department of Medical Genetics. Statistical associations were evaluated using chi-square tests, odds ratios, and logistic regression.

**Results:**

A statistically nominally significant association with prostate cancer susceptibility was observed for the *RNASEL* rs486907 polymorphism, demonstrating a potential protective effect under the dominant model in the studied population (OR = 0.53, 95% CI: 0.30–0.92, p = 0.025). In the age-adjusted logistic regression analysis, this association remained statistically significant (OR = 0.56, 95% CI: 0.32–0.99, p = 0.046), suggesting that the observed effect was independent of age. In contrast, no statistically significant associations were identified between prostate cancer risk and the investigated polymorphisms in the *KLK3*, *MSR1*, or *ELAC2* genes.

**Conclusion:**

The present study demonstrated a significant association between the *RNASEL* rs486907 polymorphism and prostate cancer susceptibility in a Hungarian cohort, suggesting a potential protective effect of this variant. These findings support the possible role of inherited genetic factors in prostate cancer development and highlight the importance of population-specific genetic association studies. Further investigations involving larger cohorts are warranted to validate these observations.

## Introduction

Prostate cancer (PCa) remains one of the most critical global health challenges in men, ranking as the most frequently diagnosed non-cutaneous malignancy and the second leading cause of cancer-related death worldwide [1]. In Hungary, PCa is the leading male malignancy, with 6,660 new cases and 1,554 deaths recorded in 2022, making it the third leading cause of cancer-specific mortality among Hungarian men [2, 3]. Established risk factors include older age, positive family history, and African ancestry [4]. Approximately 20% of patients develop high-risk tumors that may progress to metastatic disease, and PCa-related mortality remains substantial despite advances in diagnosis and treatment [5, 6].

Recent advances have highlighted the role of genetic factors in PCa susceptibility and prognosis. Although hereditary predisposition is well established, interactions between genetic and biological factors contribute to individual disease risk [7, 8]. Single nucleotide polymorphisms (SNPs) are common genetic variants [9], and genome-wide association studies (GWAS) have identified approximately 70–100 PCa-associated SNPs, supporting a polygenic model in which multiple common variants contribute to inherited disease susceptibility [8].

Among candidate susceptibility genes, *RNASEL*, *ELAC2*, and *MSR1* have been implicated in PCa risk and aggressiveness [8, 10–12]. However, associations between individual variants and PCa risk remain inconsistent across populations, potentially reflecting differences in genetic background and biological pathways including immune response, apoptosis, DNA damage response, and inflammation [8]. The *RNASEL* rs486907 variant has been associated with altered enzymatic activity, apoptosis, PCa susceptibility, and disease aggressiveness, although conflicting findings have also been reported [13, 14]. Similarly, *ELAC2* variants rs5030739 and rs17552022 and *MSR1* variants rs918 and rs1904577 have shown associations with PCa susceptibility or aggressive characteristics in some cohorts, but these findings have not been consistently replicated [10, 14].

In addition, *KLK3* rs17632542 has been associated with PCa aggressiveness, biopsy positivity, and higher-risk disease [15].

Although most PCa GWAS have been conducted in PSA-screened Western populations, Central and Eastern European cohorts remain underrepresented [16]. Therefore, the aim of the present study was to investigate the association between selected polymorphisms in *RNASEL*, *ELAC2*, *MSR1*, and *KLK3* genes and prostate cancer susceptibility in a Hungarian population, as well as their potential associations with clinicopathological parameters.

## Materials and methods

### Study population and patient selection

Data for this case-control study were collected prospectively. A total of 103 adult patients (aged over 18 years) who underwent prostate biopsy at the Department of Urology, University of Pécs, and had histopathologically confirmed prostate cancer (assigned under the International Classification of Diseases, 10th Revision [ICD-10] code C61H0) were enrolled in the study. Peripheral blood samples were collected from each patient, which were subsequently processed for genomic DNA isolation. Patients who met the diagnostic criteria but declined to provide written informed consent were excluded from the study.

The healthy control group comprised 103 age- and sex-matched men whose genomic DNA samples were obtained from the biobank of the Department of Medical Genetics, University of Pécs. These individuals were asymptomatic men who underwent routine urological screening, presenting with normal age-specific serum PSA levels (PSA <4.0 ng/mL) and negative digital rectal examination (DRE) findings, thereby minimizing the risk of including occult or latent prostate cancer cases. Due to the regional demographic characteristics of the investigated population, the study cohort consisted exclusively of Caucasian Hungarian individuals.

### Clinical data collection

Demographic and clinical data were collected using a structured questionnaire, which captured information on age, family history of malignancy, and ethnic background. Comprehensive clinical records were documented for each patient, including serum PSA levels, digital rectal examination (DRE) findings, histopathological diagnoses, and Gleason scores. Furthermore, the availability of pre-biopsy multiparametric magnetic resonance imaging (mpMRI) was recorded, along with the corresponding Prostate Imaging–Reporting and Data System (PI-RADS) scores where applicable.

### Ethical considerations

This research involving human subjects was reviewed and approved by the National Scientific Research Ethics Committee (ETT TUKEB, Budapest, Hungary; institutional approval number: BM/320893/2024). The study was conducted at the Department of Urology and the Department of Medical Genetics of the University of Pécs in strict accordance with the ethical principles of the Declaration of Helsinki (1975, as revised in 2013) and the currently operative national regulations governing human genetic research (Hungarian Law; XXI/2008). The operational and governance principles of the biobank providing the control samples were similarly approved by ETT TUKEB. Following formal genetic counseling, written informed consent was obtained from all participating individuals prior to their inclusion in the study, ensuring comprehensive protection of personal and clinical data.

### DNA extraction and genotyping

Genomic DNA was isolated from peripheral blood samples using the E.Z.N.A. Blood DNA Maxi extraction kit (OMEGA®, Bio-tek, Inc., GA, USA). The concentration and purity of the extracted DNAs were assessed using a NanoDrop 2000 spectrophotometer (Thermo Fisher Scientific, Waltham, MA, USA). PCR amplification was performed in a total volume of 50 µL containing approximately 20 ng of genomic DNA and 0.4 µM of each forward and reverse primer, without additional MgCl_2_ supplementation. The PCR cycling conditions consisted of an initial denaturation step at 95 °C for 2 min, followed by 35 cycles of denaturation at 95 °C for 30 s, annealing at a uniform temperature of 55 °C for 30 s, and extension at 72 °C for 30 s. A final extension step was carried out at 72 °C for 5 min. The resulting PCR products were then subjected to Sanger direct sequencing. Specific forward and reverse primers were custom designed for the amplification of each genetic variant; their sequences are summarized in Table 1. To determine the genotypes, Sanger direct sequencing was performed on an Applied Biosystems 3500 Genetic Analyzer (Applied Biosystems, Foster City, CA, USA) utilizing capillary electrophoresis and BigDye Terminator chemistry according to the manufacturer’s instructions. The following specific gene variants were analyzed: *MSR1* (NM_138715.3:c.*516G>A and c.1223-3957C>T), *ELAC2* (NM_018127.7:c.1893A>G; p.Thr631 = and c.1621G>A; p.Ala541Thr), *KLK3* (NM_001648.2:c.536T>C; p.Ile179Thr) and *RNASEL* (NM_021133.4:c.1385G>A; p.Arg462Gln). The sequencing call rate was 100% for the successfully analyzed samples, missing genotype frequencies were below 1%, and genotype reproducibility was confirmed via random re-sequencing of 10% of the samples with 100% concordance.

**TABLE 1:** Applied primers and PCR conditions.

Gene variant	Primer	Primer sequence (5′-3′)	Annealing temperatures
*KLK3* (NM_001648.2):c.536T>C p.(Ile179Thr)	Forward	TAT​GAG​CCT​CCT​GAA​GAA​TCG	55 °C
	Reverse	GAG​TAG​GGA​TGA​CTC​ACC​GA	
*MSR1* (NM_138715.3):c.*516G>A	Forward	AGA​TTA​CAA​AGG​CCA​AGG​GT	55 °C
	Reverse	GTG​GCA​TTT​TTG​ATC​CAC​CA	
*MSR1* (NM_138715.3):c.1223-3957C>T	Forward	ACA​ACA​GTG​GTA​CCT​CCA​AG	55 °C
	Reverse	ACA​TTC​AAC​ATG​CAA​GGA​GC	
*ELAC2* (NM_018127.7):c.1893A>G p.(Thr631 =)	Forward	ACT​TTG​GTT​CCA​GAT​GTC​CAA	55 °C
	Reverse	CAG​CTA​CAC​AAA​CCC​CAG​AG	
*RNASEL* (NM_021133.4):c.1385G>A p.(Arg462Gln)	Forward	CTC​CAC​AGA​ATA​TAC​CGC​CC	55 °C
	Reverse	GAG​AAT​TGG​GGA​TTG​GGG​AC	
*ELAC2* (NM_018127.7):c.1621G>A p.(Ala541Thr)	Forward	AAA​GGG​TTT​CAG​GGT​TCC​AT	55 °C
	Reverse	GAG​TAA​CAA​AAG​CTC​TGG​GC	

### Statistical analysis

Statistical analyses were performed using SPSS Statistics 30.0 package for Windows (SPSS Inc., Chicago, IL, USA). A dominant genetic model was applied for the analysis, which was selected due to the low frequency of rare homozygous variants in our cohort. We applied the chi-square test to compare the differences in genotype and allele distributions between patient and healthy control groups; statistical significance was verified using chi-square tests where appropriate, and the p-values have been updated accordingly. A *p* ≤ 0.05 value was considered as statistically significant. The genotype and allele frequency distributions, together with the corresponding ORs, 95% CIs, and p-values, are presented in Table 2.

**TABLE 2:** Genotype and allele frequencies of the investigated polymorphisms in prostate cancer patients and healthy controls, including dominant association analysis.

Gene	Polymorphism	Genotype	Patients, n (%)	Controls, n (%)	OR (95% CI)	*P*
*KLK3*	c.536T>C	TT	91 (88.3%)	90 (87.4%)	​	​
		TC	12 (11.7%)	12 (11.7%)	​	​
		CC	0 (0%)	1 (1%)	​	​
		C allele frequency	5.8%	6.8%	0.91 (0.40–2.11)	0.831
*MSR1*	c.*516G>A	GG	93 (90.3%)	90 (87.4%)	​	​
		GA	10 (9.7%)	10 (9.7%)	​	​
		AA	0 (0%)	3 (2.9%)	​	​
		A allele frequency	4.9%	7.8%	0.74 (0.31–1.78)	0.507
*MSR1*	c.1223-3957C>T	CC	2 (1.9%)	2 (1.9%)	​	​
		CT	20 (19.4%)	26 (25.2%)	​	​
		TT	81 (78.6%)	75 (72.8%)	​	​
		T allele frequency	88.3%	85.4%	1.00 (0.14–7.24)	1.000
*ELAC2*	c.1893A>G	AA	75 (72.8%)	75 (72.8%)	​	​
		AG	26 (25.2%)	24 (23.3%)	​	​
		GG	2 (1.9%)	4 (3.9%)	​	​
		G allele frequency	14.6%	15.5%	1.00 (0.54–1.85)	1.000
*ELAC2*	c.1621G>A	GG	96 (93.2%)	99 (96.1%)	​	​
		GA	7 (6.8%)	4 (3.9%)	​	​
		AA	0 (0%)	0 (0%)	​	​
		A allele frequency	3.4%	1.9%	1.81 (0.51–6.36)	0.353
*RNASEL*	c.1385G>A	GG	53 (51.5%)	37 (35.9%)	​	​
		GA	27 (26.2%)	48 (46.6%)	​	​
		AA	23 (22.3%)	18 (17.5%)	​	​
		A allele frequency	35.4%	40.8%	0.53 (0.30–0.92)	**0.025**

Dominant model: heterozygous + variant homozygous genotypes vs. reference homozygous genotype.

Bold indicates nominal statistical significance (p < 0.05).

Bold: Statistically significant P value.

## Results

**FIGURE 1:**
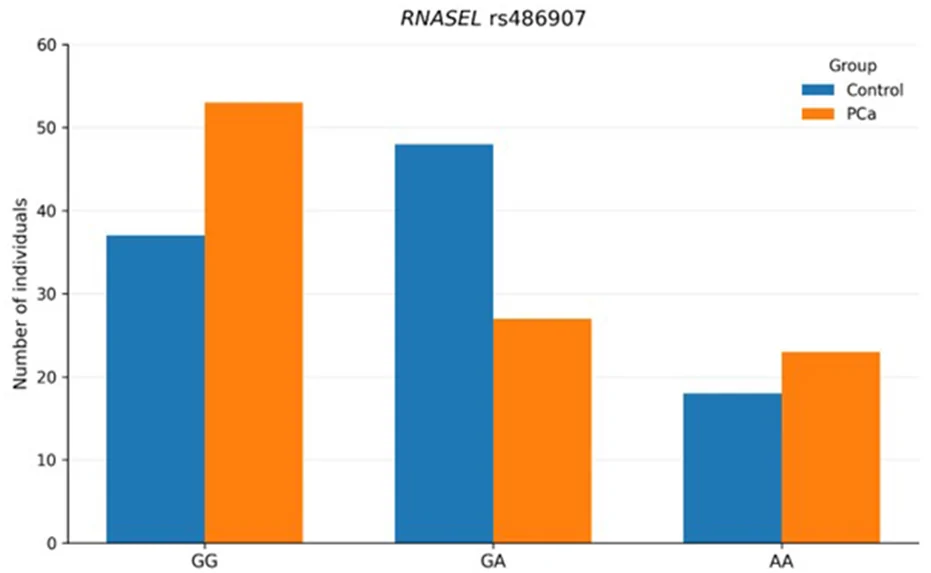
Distribution of *RNASEL* rs486907 genotypes in PCa patients and controls.

This case-control study included 103 histologically confirmed prostate cancer patients and 103 healthy controls. The mean age of the patients was 68.9 ± 6.2 years, while the mean age of healthy controls was 67.2 ± 5.8 years. Based on serum PSA levels, 50 patients (48.5%) had PSA values ≤ 10 ng/mL, 21 patients (20.4%) had PSA levels between 10 and 20 ng/mL, while 32 patients (31.1%) presented with PSA levels >20 ng/mL. A Gleason score <7 was observed in 22 patients (21.4%), whereas 81 patients (78.6%) had a Gleason score ≥7. According to the EAU guideline–based clinical risk stratification using both PSA level and Gleason score, 50 patients (48.5%) were classified as low-risk, 21 patients (20.4%) as intermediate-risk, and 32 patients (31.1%) as high-risk disease (Table 3). During the genetic analysis, genotype and allele frequencies of six selected polymorphisms in the *MSR1*, *ELAC2*, *KLK3* and *RNASEL* genes were determined in both studied groups. These distribution data, along with the corresponding odds ratios (OR), 95% confidence intervals (CI), and p-values, are summarized in Table 2. All investigated polymorphisms were in Hardy–Weinberg equilibrium in the control group. Among the evaluated loci, *RNASEL* c.1385G>A; p.Arg462Gln (rs486907) was the only variant to show a statistically significant association with prostate cancer risk. The GG homozygous normal genotype was more frequent among prostate cancer patients (51.5%) compared to controls (35.9%), whereas the heterozygous GA genotype occurred more frequently in the control group (46.6% vs. 26.2%). The A allele frequency was 35.4% in patients and 40.8% in controls. Due to the exploratory nature of these 103 cases versus 103 control study, the reported associations are nominally significant, as multiple testing corrections were omitted. Descriptive comparison with external reference datasets was performed to position the current cohort within a wider population context (Table 4). The *RNASEL* rs486907 A-allele frequency among Hungarian controls (0.408) was broadly consistent with the European reference values from the 1000 Genomes Project (0.371). In our prostate cancer cohort, the A-allele frequency stood at 0.354. For comparison, the global meta-analysis by Zuo et al. yielded frequencies of 0.303 for affected individuals and 0.290 for healthy controls. Association analysis demonstrated a statistically significant protective effect of the variant under the dominant model in the investigated population (OR = 0.53; 95% CI: 0.30–0.92; p = 0.025). Furthermore, in age-adjusted logistic regression analysis, this protective association of the *RNASEL* rs486907 dominant model remained statistically significant (OR = 0.56, p = 0.046), suggesting that the observed association was independent of age. The distribution of *RNASEL* rs486907 genotypes in prostate cancer patients and controls is illustrated in Figure 1. No statistically significant associations with prostate cancer susceptibility were observed for the genetic variants within the *MSR1*, *ELAC2*, and *KLK3* genes.

**TABLE 3:** Clinical characteristics and risk stratification of prostate cancer patients.

Variable	PCa patients (n = 103)
Age (years)	68.9 ± 6.2
Low-risk (PSA + Gleason based)	50 (48.5%)
Intermediate-risk (PSA + Gleason based)	21 (20.4%)
High-risk (PSA + Gleason based)	32 (31.1%)

**TABLE 4:** Comparison of the *RNASEL* rs486907 variant A allele frequencies across different populations.

Population/study cohort	Population group	Variant allele frequency	References
Healthy controls	Hungarian	0.408 (40.8%)	Present study
PCa cases	Hungarian	0.354 (35.4%)	Present study
1000 genomes – European	EUR	0.371 (37.1%)	1000 genomes Project [17]
1000 genomes – East Asian	EAS	0.242 (24.2%)	1000 genomes Project [17]
1000 genomes – African	AFR	0.067 (6.7%)	1000 genomes Project [17]
1000 genomes – American	AMR	0.223 (22.3%)	1000 genomes Project [17]
1000 genomes – South Asian	SAS	0.302 (30.2%)	1000 genomes Project [17]
Meta-analysis – PCa cases	Pooled PCa cases	0.303 (30.3%)	Zuo et al. [18]
Meta-analysis – controls	Pooled controls	0.290 (29%)	Zuo et al. [18]

## Discussion

The present study investigated the association between selected polymorphisms in *RNASEL*, *ELAC2*, *MSR1*, and *KLK3* genes and prostate cancer susceptibility in a Hungarian population. Among the investigated variants, only the *RNASEL* rs486907 polymorphism demonstrated a statistically nominally significant association with prostate cancer risk, suggesting a potential protective effect in the studied cohort.

*RNASEL* is considered a biologically plausible candidate gene in prostate cancer susceptibility due to its role in interferon-mediated antiviral defense, apoptosis, and inflammatory signaling pathways [11]. The rs486907 (p.Arg462Gln) polymorphism has previously been associated with altered RNASEL enzymatic activity and impaired apoptotic function [19]. Several studies reported associations between this variant and increased prostate cancer susceptibility or more aggressive disease phenotypes, including higher Gleason score and familial prostate cancer [10, 14]. However, the available literature remains inconsistent, as other investigations described either no association or even protective effects of specific *RNASEL* variants [13, 20]. These contradictory findings suggest substantial population-dependent variability and support the hypothesis that inherited susceptibility to prostate cancer may differ between ethnic and geographic populations. To place these findings in a broader population context, the *RNASEL* rs486907 A-allele frequencies observed in the present cohort were compared descriptively with data from the 1000 Genomes Project and the meta-analysis by Zuo et al. (Table 4). The A-allele frequency in the Hungarian control group (0.408) was broadly comparable to that reported for the European reference population (0.371). The corresponding frequency among prostate cancer patients was 0.354. In the pooled meta-analysis by Zuo et al., the A-allele frequencies were 0.303 among prostate cancer cases and 0.290 among controls [10, 17, 18]. This discrepancy suggests that the association between *RNASEL* rs486907 and prostate cancer risk may vary across populations and study cohorts. As no formal statistical comparison with external reference populations was performed, these differences should be interpreted descriptively.

Mechanistically, the *RNASEL* rs486907 (p.Arg462Gln) variant alters protein function and leads to a threefold reduction in enzymatic activity [19, 21]. While compromised *RNASEL* activity is traditionally linked to impaired apoptosis and viral clearance [21, 22], it is hypothesized that a dampened immune response may conversely mitigate chronic intraprostatic inflammation.

Given that chronic inflammation is an important contributor to prostate carcinogenesis [23, 24], the reduced enzymatic activity of the Gln462 variant might theoretically exert a protective effect in specific genetic backgrounds by suppressing tumor-promoting inflammatory microenvironments. However, this proposed protective mechanism remains purely hypothetical and speculative, as it has not been directly demonstrated or functionally validated for the rs486907 variant.

The protective association observed in the present study is consistent with previous reports suggesting that certain *RNASEL* variants or haplotypes may reduce prostate cancer risk in specific populations [13, 20]. Differences in allele frequencies, linkage disequilibrium patterns, environmental exposures, and gene–gene interactions may contribute to these heterogeneous findings. Furthermore, prostate cancer is considered a polygenic and multifactorial disease in which the individual effect of a single polymorphism is likely modest and influenced by additional genetic and non-genetic factors [25].

No statistically significant associations were observed for the investigated *ELAC2*, *MSR1*, or *KLK3* polymorphisms. Although previous studies reported potential associations between these variants and prostate cancer susceptibility or aggressiveness, the findings have not been consistently reproducible across different cohorts [10, 14]. The lack of significant associations in the present study may partially be explained by population-specific genetic differences and the relatively limited sample size. Notably, our PCa cohort was characterized by a high proportion of aggressive disease, with 78.6% of patients presenting with a Gleason score ≥7 and 31.1% demonstrating high-risk clinical features according to EAU stratification [26]. The lack of statistically significant associations for the *ELAC2* [27]*, MSR1* [14], and *KLK3* [28] variants within our study could potentially be attributed to this clinical skewness, as these markers might exert different risk profiles in low-grade or indolent prostate tumors, which were underrepresented in our sample.

The present study has several limitations. First, the sample size was relatively limited, which may reduce statistical power, complicate the identification of variants with small effect sizes, and increase the risk of false-negative findings. Accordingly, further stratification into clinical subgroups, such as by Gleason score or PSA categories, was not performed, as sub-analyzing smaller groups would have further reduced statistical power and increased the risk of spurious associations. Second, this was a single-center study involving a Hungarian population; therefore, the results may not be generalizable to other ethnic groups. Third, correction for multiple testing was not applied due to the exploratory nature of the study and the limited cohort size. Consequently, the observed borderline significant associations must be interpreted with caution and require independent validation in larger, well-powered cohorts. In addition, detailed clinical data were not available for the control group, limiting further comparative analyses.

Despite these limitations, the study also has important strengths. All prostate cancer cases were histologically confirmed, the investigated cohort was clinically well characterized, and genotyping was performed using PCR-based Sanger sequencing, providing reliable genetic analysis. Furthermore, data regarding prostate cancer susceptibility polymorphisms in Central and Eastern European populations remain limited, highlighting the potential relevance of the present findings.

Importantly, the protective association of the *RNASEL* rs486907 variant remained statistically significant even after adjusting for age in the multivariate logistic regression analysis (OR = 0.56, 95% CI: 0.32–0.99, p = 0.046). Since advanced age is the strongest non-modifiable risk factor for PCa [29], this age-independent persistence is noteworthy. However, given the limited sample size and the fact that this finding is based on nominal significance, it should be treated as exploratory rather than a definitive genetic predisposition. Moreover, because its individual effect size is too small for direct, standalone clinical risk prediction or immediate therapeutic application, we instead highlight its value as a potential candidate for future, larger-scale Central European polygenic risk models. Furthermore, the adherence of all investigated loci to the Hardy–Weinberg equilibrium in the control group supports the quality of genotyping and indicates no apparent deviation that would raise concerns regarding genotyping errors or major population stratification [30].

## Data Availability

The raw data supporting the conclusions of this article will be made available by the authors, without undue reservation.
